# Modeling *Salmonella* Spread in Broiler Production: Identifying Determinants and Control Strategies

**DOI:** 10.3389/fvets.2020.00564

**Published:** 2020-08-25

**Authors:** Pedro Celso Machado Junior, Chanjin Chung, Amy Hagerman

**Affiliations:** Department of Agricultural Economics, Oklahoma State University, Stillwater, OK, United States

**Keywords:** *Salmonella*, broiler chicken, risk analysis, Bayesian hierarchical model, principal-agent problem

## Abstract

The presence of *Salmonella* spp. in broiler production is a food safety concern as the bacterium can be transmitted to humans via contaminated meat and derived products. Salmonella detection in litter at the pre-slaughter period has been linked to increased odds of contaminated broiler carcasses and meat derived products. To determine risk factors related to farm and broiler house characteristics and management practices, this study uses a unique longitudinal data set from a Brazilian integrated broiler enterprise, which contains official results of *Salmonella* spp. isolation from drag swabs collected at the end of the grow-out period. A Bayesian hierarchical spatio-temporal model found significant spatial and time influence on the odds of isolating *Salmonella* spp. from litter as well as significant effects from the size of a broiler house, total housing area per farm, type of broiler house, and number of litter recycles. Results indicate that recycling litter beyond 6 rearing cycles significantly increased the odds of isolating Salmonella before slaughter, and the bacterium was more likely to persist in conventional broiler houses, compared to broiler houses with controlled environment. Evidence of a potential principal-agent problem was also found in setting strategies to control the bacterium from litter, which suggests strong incentives to adopt the strategies aiming to reduce prevalence of the bacterium in the integrated enterprise. Our findings could be used to develop alternative measures to reduce the risk of persistence of the bacterium in the broiler production chain.

## Introduction

Poultry meat is currently the world's most consumed and affordable meat type among animal-source. For the coming decade, per capita consumption of poultry meat is expected to increase by 5.5% worldwide, highlighting the importance of this commodity to food security, and protein availability (1). However, consumption of contaminated poultry meat was reported to cause 20.6% of foodborne diseases in the US between 1998 and 2008, among which *Salmonella* spp. was one of the main etiological agents (2).

Salmonella is a natural inhabitant of the gastrointestinal tract (GIT) of birds and can be introduced into the production system through several ways like contaminated feed or water, live vectors, contaminated litter, and even humans via contaminated boots or tools (3). Therefore, to effectively control the bacteria within a poultry enterprise, many critical control points involving different production stages must be properly observed from the parent stock, feed production, transportation, on-farm interventions and finally at the processing plant. In this regard, major efforts must be directed to reducing the bacterial load entering the processing plant, as cross contamination is a major source of bacterial detection at this level (4–6).

Healthy poultry frequently harbor Salmonella, and the transmission of the bacteria from meat and contaminated eggs is suggested as the main risk factor for human contamination. Effort involving surveillance, biosecurity, and vaccination has been related to substantial reduction in salmonellosis cases in Europe, highlighting the importance of adopting effective control measures in poultry and egg production, focusing primarily on serotypes related to human diseases like *Salmonella Enteritidis* and *Salmonella Typhimurium* (7).

Several risk analysis and modeling frameworks were performed to identify and suggest control measures for the risk of foodborne disease caused by Salmonella from broiler chicken (8–10), layers (11), pigs (12–14), and dairy cattle (15, 16).

Hygiene practices targeting bacterial elimination and prevention of contamination are constantly found to be interventions providing the greatest benefits in reducing prevalence at both production sites and processing plants (17, 18).

Epidemiological models have been used to model the spread of salmonella in many livestock production systems (12, 14–16, 19, 20). Most of these studies, however, have not accounted for dynamic decisions within the production system and are unable to estimate important parameters related to transmission and prevalence of diseases. The studies tend to rely heavily on assumptions, which makes the results fragile from applied perspectives. Furthermore, the lack of information from field controlled trials or field observations collected in a consistent manner is a major drawback when attempting to model real-life scenarios (21), underscoring the need to incorporate field data into a modeling framework. Such a task, however, is not trivial once not all firms keep a consistent scheme of data collection or are willing to disclose information.

When it comes to modeling the spread of salmonella within a broiler enterprise, it is crucial to have data of bacterial presence from different stages of production. The data are traceable across different production units and are repeated measurements from different farms throughout the processing plant. Such information allows the estimation of the likelihood of detecting the bacteria as a function of determined risk factors, aiming to further improve the control and ultimately eradicate the infection with evidence-based decision making. This information can be further applied to a commonly used epidemiological model to assess the optimal control measures given a set of available alternatives applicable to the specific enterprise.

However, when dealing with repeated measurements across time, temporal, and spatial autocorrelation must be accounted for to identify risk factors related to the occurrence of salmonella. It is intuitive that a poultry house that is positive to salmonella infection is more likely to remain positive if disinfection protocols are not properly applied, which will be translated into time autocorrelation. Similarly, a poultry house that is located closer to one that is positive for *Salmonella* spp. is more likely to be contaminated by vectors or fomites than poultry houses that are more spatially isolated. This neighborhood effect will ultimately be a cause of spatial autocorrelation.

The presence of spatial and temporal autocorrelation are problematic when fitting logistic regressions as the assumption of independent and identically distributed (i.i.d.) errors is violated, which especially affects the statistical significance of risk factor estimates. Furthermore, when evaluating risk factors for a determined biological agent, it may be of interest to identify and account for spatial patterns across time. Most studies evaluating risk factors for the presence of *Salmonella* spp. in livestock tend to consider random effects attributed to the farm or one specific region (6, 8, 22, 23). Few studies use longitudinal data, and most studies do not account explicitly for spatial autocorrelation.

Our study defines risk factors among farm characteristics and management practices consistently controlled by an integrated broiler enterprise related to the isolation of *Salmonella* spp. from litter in the grow-out period. We estimate a spatio-temporal Bayesian hierarchical binomial logistic regression model using field data of *Salmonella* spp. isolation in broiler houses from different farms in the south region of Brazil. The model captures the spatial and temporal patterns in Salmonella occurrence via random effects, while setting conditional autoregressive (CAR) priors. The probability of salmonella detection is then defined to be a function of covariates pertaining to consistently recorded farm characteristics and practices and the random spatio-temporal effects. The article contributes to literature by determining the effect of farm characteristics (e.g., size of broiler house and type of broiler house), as well as management practices (e.g., litter recycle) on the probability of isolating *Salmonella* spp. from litter, while explicitly accounting for spatial and temporal sources of variations.

Model estimates are used to calculate odds ratio for each of relevant risk factors to identify determinants of *Salmonella* spp. spread and draw control strategies for policy implications. We also discuss optimal control measures from estimated parameters and expected probabilities and show the effect of interventions related to litter recycles on the enterprise expected return using a partial budget and net present value (NPV).

## Materials and Methods

### Data

The dataset comprises results of isolation of *Salmonella* spp. in litter of 417 different broiler flocks, collected from 139 broiler houses serving a vertically integrated company located in a south region of Brazil. The data of *Salmonella* spp. isolation were recorded from three consecutive flocks of each broiler house, accounting for a total evaluation time of 195 days. Drag swabs samples were collected from the litter of every broiler house 15 days before slaughter (average rearing time was 45 days). The collection was made by trained field technicians following standard protocols and analyzed by an accredited laboratory according to the recommendations described in the Ordinance 126 of November 3rd, 1995 (24), and following the program established by the Brazilian Ministry of Agriculture to control *Salmonella* spp. in broiler chickens and turkeys (25).

Briefly, the sampling procedure consists in dragging an assembly of at least three separate moistened 10 cm × 10 cm surgical gauze swabs, attached to a string stapled to a wooden spatula over the litter along the length of the broiler house, using the water and feeder lines as sectioning guides (26). The samples are then placed in transport media and immediately sent for analysis.

Spatial location of each broiler house was recorded using global positioning system (GPS) coordinates. The coordinates were then used to identify neighbors of every poultry house by Euclidean distance, using a circle of 20 km from each broiler location as a cutoff point. Under this specification, the obtained neighborhood matrix reveals an average number of links of 33.71 for each broiler house. Three broiler houses were the most connected with 63 links, while two were the least connected with only 1 link. Average link distance was 11.45 km^1^, and median distance was 11.89 km.

Table 1 summarizes the farm characteristics adopted as covariates, used by the enterprise to characterize broiler houses, farms, and the practice of recycling litter. Size of broiler house relates to the total area of the broiler house in thousands of square meters. This is a continuous variable and ranges from 900 m^2^ to 5,400 m^2^, with mean value across all farms of 2,230 m^2^. Number of broiler houses per farm was also a continuous variable ranging from one to four, with mean 1.52, which records the number of different broiler houses under the same farm unit. A dummy variable to indicate whether the broiler house was located on a farm with a single broiler house (0) or on a farm with multiple broiler houses (1) was included to identify possible management effects, as multiple broiler house farms tend to be more specialized. Total housing size is a continuous variable that the enterprise uses to measure the total broiler production area, in square meters, of the farm and is obtained by summing the areas of broiler houses in that particular farm. This variable ranges from 1,200 m^2^ to 14,400 m^2^, with mean value of 3,940 m^2^.

**Table 1:** Description of farm characteristics and practices adopted as covariates.

**Covariate**	**Type**	**Code**	**Description**
Size of broiler house (1,000 m^2^)	continuous	House size	Min = 0.90, average = 2.23, max = 5.40
Number of broiler houses/farm	continuous	N_houses	Min = 1.00, average = 1.52, max = 4
Single house	categorical	single	Dummy variable taking the value of 0 if farm has only one broiler house and 1 if farm has 2 or more broiler houses
Total housing size (1,000 m^2^)	continuous	Total housing size	min = 1.20, average = 3.94, max = 14.40
Type of broiler house	categorical with three levels	Type1, Type2, Type3	1-Old building with curtains, 2-New building with curtains, 3-New building with climate control
Number of litter recycles	continuous	Litter_use	min = 1.00, average = 5.72, max = 22.00
Presence of livestock	categorical	Livestock	1 if present, 0 otherwise
Presence of dogs	categorical	Dogs	1 if present, 0 otherwise
Presence of crop areas	categorical	Crops	1 if present, 0 otherwise

Type of broiler house is a categorical variable used to characterize broiler houses across farms and is related to the structure and age of the building, type of equipment and isolation. Type 1 and type 2 broiler houses are conventional houses, with lateral curtains for insulation and ventilation, sprinklers, fans, automatic feeders, and drinkers. Main difference between types 1 and 2 houses relates to the age of the building, which is >5 years for type 1 and <5 years for type 2. Type 3 houses are those with negative pressure and controlled environment, with evaporative panels, automatic drinkers, and feeders.

The number of litter recycle indicates the number of times the litter used in one flock is treated in the between flock period and used on the next flock, with little or no addition of new litter. The average number of recycles is 5.72, ranging from 1 to 22 recycles. Wood shavings are used as bedding material in this enterprise and compose the litter. Other variables recorded are categorical and relate to the presence (1) or absence (0) of livestock, dogs or crop areas in the farm where the broiler house is located.

Out of the 139 evaluated broiler houses, 45, 74, and 77 were found to be positive for *Salmonella* spp. at the end of the first, second and third rearing cycles, accounting for an estimated raw prevalence of 32.37, 53.32, and 55.39%, respectively.

### Model Specification

Each of the broiler houses in this study is considered a unique spatial unit k, with k = 1, …, K = 139, defined by a GPS location. Data on presence or absence of *Salmonella* spp. at the end of each t = 1,.., T = 3 rearing periods is recorded for every unit. Denoting by θ_*kt*_, the probability of detecting *Salmonella* spp. in litter of the k-th broiler house at time *t*, a Bayesian hierarchical logit model is described as:

(1) $$\begin{array}{r} \ln\left(\frac{\theta_{kt}}{1-\theta_{kt}}\right)=\;{{\text{X}}_{kt}}^{\prime }\beta +\varphi_{kt}+\delta_{t}. \end{array}$$

The logit probabilities of *Salmonella* spp. detection are modeled as a liner combination of a *p* × 1 vector of covariates **X**_*kt*_, and spatial φ_*kt*_, and temporal δ_*t*_ random effects, where *p* represents covariates described in Table 1, and their respective vector of regression parameters **β**.

It is assumed that **β** follows a multivariate normal distribution and a diffuse multivariate normal prior distribution is specified: **β** ~ *N*(0, 1, 000**I**), where **I**_*p* × *p*_ is the identity matrix.

The spatial random effect φ_*kt*_ and temporal random effect δ_*t*_ model spatial and temporal trends and autocorrelation in the data after accounting for the covariate effects. Spatial autocorrelation is controlled by a symmetric K × K neighborhood weight matrix ***W*** = (*w*_*kj*_), where *w*_*kj*_ represents spatial closeness between spatial units (*S*_*k*_, *S*_*j*_), and *w*_*kj*_ is non-zero if they share a common border and zero otherwise, and *w*_*kk*_ = 0 for all k. Temporal autocorrelation is controlled by a binary N × N temporal neighborhood matrix ***D*** = (*d*_*tj*_), where *d*_*tj*_ = 1 if | j–t | = 1 and *d*_*tj*_ = 0 otherwise.

It is specified as:

(2) $$\begin{array}{r} \varphi_{t}\;\sim N\left(0,\;\tau_{t}^{2}Q{\left(W,\rho_{S}\right)}^{-1}\right)for\;t=\;1,\ldots ,N, \end{array}$$

where φ_*t*_ = (φ_1*t*_, …, φ_*kt*_) is the vector of all spatial random effects at period *t*, and the spatial autocorrelation in the data is modeled by the matrix ***Q***(***W***, ρ_*S*_) = [ρ_*S*_(*diag*(***W*1**) − **W**) + (1−ρ_*S*_)**I**], where 1 is a K × 1 vector of 1′s, so that diag (W1) is a diagonal matrix with diagonal elements equal to the row sums of W. W and **I**_*K* × *K*_ are the neighborhood and identity matrices, respectively. The full conditional specification of φ_*kt*_ is then:

(3) $$\begin{array}{r} \varphi_{kt}|\varphi_{-kt},W,\rho ,\;\tau_{t}^{2}\;\sim N(\frac{\rho_{S}\overset{K}{\underset{j=1}{\sum }}w_{kj}\varphi_{jt}}{\rho_{S}\overset{K}{\underset{j=1}{\sum }}w_{kj}+1-\rho_{S}}\;,\;\\ \;\frac{\tau_{t}^{2}}{\rho_{S}\overset{K}{\underset{j=1}{\sum }}w_{kj}+1-\rho_{S}}\;), \end{array}$$

where **φ**_−*kt*_ = (φ_1, *t*_, …, φ_*k*−1,*t*_, φ_*k*+1,*t*_, …, φ_*K, t*_).ρ_*S*_ measures the strength of spatial autocorrelation and is assumed to be constant over time, as variances $\tau_{t}^{2}$ are allowed to change temporally, thus, capturing changes on spatial variability.

For the temporal random effect, it is specified as:

(4) $$\begin{array}{r} \delta_{t}|\delta_{-t},\;D\;\sim N(\frac{\rho_{T}\overset{N}{\underset{j=1}{\sum }}d_{tj}\delta_{j}}{\rho_{T}\overset{N}{\underset{j=1}{\sum }}d_{tj}+1-\rho_{T}},\\ \;\frac{\tau_{T}^{2}}{\rho_{T}\overset{N}{\underset{j=1}{\sum }}d_{tj}+1-\rho_{T}}\;), \end{array}$$

where **δ** = (δ_1_, …, δ_*N*_). ρ_*T*_ measures the strength of temporal autocorrelation and the temporal random effects capture the overall temporal trend in the probability of isolating *Salmonella* spp. in litter across all broiler houses. The spatial random effects model was proposed by Leroux et al. (27), while the temporal random effects were described in Besag et al. (28).

Priors are specified for parameters from Equations (3) and (4) as:

(5) $$\begin{array}{r} \tau_{1}^{2},\ldots ,\tau_{N}^{2},\;\tau_{T}^{2},\;\sim \;Inverse-Gamma\left(1,0.01\;\right)\\ \;\rho_{S},\rho_{T}\;\sim \;Uniform\left(0,1\right). \end{array}$$

The distributions and parameter values in Equation (5) are chosen because they provide flat and conjugate priors, as described in Lee et al. (29). Sampling from the posterior distributions is obtained using Markov Chain Monte Carlo simulation with Gibbs sampling and Metropolis-Hastings algorithms. Computations are made in the R software, using package CARBayesST.

Spatial dependence is evaluated by first fitting the Bayesian hierarchical model specified in equation (1) without including random effects. Residuals are recovered and used to compute Moran's I (30) statistics, performing permutation tests on the residuals separately for each year. The null hypothesis tested is of no spatial autocorrelation and the alternative hypothesis is of positive spatial autocorrelation. Temporal autocorrelation at lag 1 was also computed from the residuals using a Lagrange multiplier test for serial correlation (31) across all locations (null hypothesis of no serial autocorrelation, and alternative hypothesis of autocorrelation of order 1).

To select relevant covariates, we first estimate equation (1) including all variables in Table 1 with relevant interactions. These covariates are included to represent the standard poultry environment in Brazil to incorporate risk factors that are frequently examined in previous studies (23, 32, 33). After estimating the model, variables with insignificant estimates were removed from model specification. The model was then re-estimated without the insignificant covariates. This exercise was done iteratively until the final model was obtained. The Deviance Information Criterion (DIC), an information criterion that accounts for model goodness of fit while penalizing complexity (34) was also used to compare different specifications. DIC can be easily calculated from posterior samples and is preferred over other information criteria (like Akaike information criterion and Schwarz-Bayes information criterion) for being more appropriate in hierarchical models. This model selection approach is commonly applied in the epidemiological literature (6, 23, 35).

Model estimates were obtained after generation of 200,000 samples, following a burn-in period of 50,000 samples. Convergence for the chain of each posterior distribution was assessed to have been reached using Geweke's statistics (36), which is based on the normal approximation and measures the sampled mean value of the first 10% of the chain as compared to the last 50%. If the calculated statistic is >|1.96|, there is evidence of poor convergence, as calculated sample means at the beginning of the chain are substantially different than calculated mean at the end of the chain. Subsequently, 150,000 samples were generated, where every 10th draw was stored and the rest discarded to remove the autocorrelation, leaving inference based on 15,000 samples.

### Economic Analysis

We use production cost and revenue estimates reported by Miele et al. (37) for an integrated broiler enterprise in the studied region and provide an example of how the model estimates and Odds Ratios can be translated into economic terms. The costs of litter replacement per flock and total labor costs as a proportion of total working costs^2^, and expected return per flock over total capital costs^3^ are calculated considering a 6% annual return rate, following Miele et al. (37). We compare the impact of positive flocks on expected return over total working costs.

The expected return is calculated adopting a baseline scenario for each type of broiler house, assuming that litter will be completely replaced after six cycles, a common practice considered for the expected return and working costs calculation per flock (37). On the integrated system, the cost of litter replacement is a responsibility of the producers. Therefore, to increase return, there may be an incentive to recycle litter beyond the recommended number of cycles to reduce working costs and therefore increase profit.

Assuming that litter recycles is the only risk factor responsible for *Salmonella* spp. transmission and persistence, we want to evaluate how the dynamics of potential cost reduction may affect incentives to recycle litter, considering a 2-year (12 rearing cycles) interval.

We assume producers are allowed to choose between two possible strategies of litter replacement: strategy 1 (baseline) is to follow the recommendation of the integrator and recycle litter for six rearing cycles, than replace it completely, and strategy 2 (baseline+additional recycle) is to recycle litter beyond six rearing cycles, replacing it completely only after 12 rearing cycles. We assume that positive flocks will have a 40% penalty reduction on total return^4^, which will be transferred to the farmer according to the proportion of produced positive flocks only if litter is recycled more than 6 times.

The problem faced by the producer may be defined by:

(6) $$\begin{array}{r} {\text{max}}_{st}\;\overset{12}{\underset{t=1}{\sum }}\frac{{NR\left(st\right)}_{t}}{{\left(1+r\right)}^{t}} \end{array}$$

Where *st* is a discrete choice related to the strategy to be chosen of follow the recommendation of the integrator, recycling litter only 6 times, then replacing it and follow with an additional six recycles (strategy 1-baseline), or recycling litter more than 6 times (strategy 2-baseline + additional recycle), *NR*(*st*)_*t*_ is the net return at cycle *t* obtained after following each litter recycle strategy for each of the broiler houses, *r* =*1 %* is the discount rate.

We see that for *t* = *1* to *t* = *6*, *NR*(*st*)_*t*_ is the same for both strategies within each broiler house, as no penalties are applied for positive flocks, while for *t* = *7* to *t* = *12*, *NR*(2)_*t*_ can be calculated as follows:

(7) $$\begin{array}{r} {NR\left(2\right)}_{t}=ER_{t}\times \left(1-\frac{1}{2}\left({\hat{\theta_{t}}}_{\frac{N}{2}}+{\hat{\theta_{t}}}_{1+\frac{N}{2}}\right)\times 0.4\right), \end{array}$$

Where *ER*_*t*_ is the expected revenue (in %) at time *t* obtained from Miele et al. (37), ${\hat{\theta_{t}}}_{\text{N}}=\;\frac{exp\left({\text{X}}_{t}^{T}\beta \right)}{1+exp\left({\text{X}}_{t}^{T}\beta \right)}$ is the ordered *N-th* draw of the posterior density of the estimated probability of isolating *Salmonella* spp. when the covariates **X** are litter recycles (Litter_use and Litter_use^2^) and type of broiler house (defined in Table 4), with *N* = *1,…,15,000*.

We present the calculation of cost per flock of litter replacement for each type of broiler house, as well as the expected return per flock and expected loss from positive flocks for recycles >6 periods. We then show calculations of net present value, obtained from Equation (6) for both strategies and discuss its implications.

## Results

Table 2 shows the presence of positive spatial autocorrelation in second and third rearing cycles based on Moran's *I*-test statistics (Table 2), confirming the adequacy of a spatial model. Temporal autocorrelation was also detected (0.22 on average across all locations—not shown in tables), suggesting the presence of positive autocorrelation at lag 1.

**Table 2:** Test results for spatial autocorrelation at each rearing cycle (time period).

**Rearing cycle**	**Observed rank**	**Test statistic^**a**^**	***p*-value**
1	1,252	−0.023	0.874
2	1,243	0.024**	0.048
3	9,513	0.027**	0.033

a *Moran's I test statistic was obtained after 10,000 simulations. H_0_ = no spatial autocorrelation, H_1_ = positive spatial autocorrelation*.

** *Denote significance at the 5% level*.

The Bayesian posterior medians and 95% credible intervals for equation (1), reported in Table 3, show that all covariates except the number of broiler houses per farm and the dummy variable indicating a single or multiple broiler house per farm, presence of livestock, dogs, or crops significantly affected the probability of isolating *Salmonella* spp. from litter. In a Bayesian setting, the posterior density is used to asses if an independent variable has a non-zero effect over the response by defining the 2.5 and 97.5% limits for the distribution, which is normally defined as the 95% credible interval. If this credible interval does not contain zero, then the effect of the independent variable may be understood as “significant” or non-zero. Interactions between type of broiler house and each of the numerical variables were also evaluated and found to be insignificant (results not shown for brevity)^5^.

**Table 3:** Bayesian hierarchical logit posterior medians and credible intervals including all covariates described in Table 1^a^.

**Variable**	**Parameter**	**Median**	**2.5%**	**97.5%**	**Geweke^**b**^**
Intercept	β_0_	−2.823	−4.852	−0.608	0.3
House size	β_1_	3.043	1.340	4.651	−0.6
House size^2^	β_2_	−0.314	−0.557	−0.058	0.7
Litter_use	β_3_	−0.209	−0.452	0.006	1.9
Litter_use^2^	β_4_	0.017	0.001	0.036	−1.9
Total housing size	β_5_	−0.349	−0.536	−0.185	0.3
Type2	β_6_	−1.169	−2.172	−0.129	0.3
Type3	β_7_	−1.890	−3.186	−0.457	0.4
N_houses	β_8_	0.392	−0.336	1.094	−0.6
Single	β_9_	−0.261	−1.168	0.621	0.6
Livestock	β_10_	−0.723	−1.597	0.113	0.2
Dogs	β_11_	0.555	−0.231	1.364	−0.3
Crops	β_12_	0.000	−0.661	0.648	0.8
	DIC^c^ = 535.97				

*Dependent variable is the isolation of Salmonella spp. in litter (n = 417)*.

a *Random effects estimates are not shown*.

b *Geweke diagnostic: values lower than |1.96| suggest good mixing of the chains*.

c *Deviance information criterion*.

To allow for non-linear responses of the numeric variables, a quadratic term was included, and was found to be significantly different from zero only for size of broiler houses and litter reutilization as shown in Table 3^6^.

Table 3 also shows the posterior median and credible intervals for all covariates listed in Table 1, as well as Geweke statistics. The effect of number of broiler houses per farm (N_houses) and whether the farm has one or multiple broiler houses (single) was not different from zero. The same was true for the presence of livestock, dogs, or crops. Geweke statistics for all posterior distributions reveals good mixing of samples and provide evidence of converge of the chains. Random effect estimates are not shown in Table 3 for brevity but were also accounted for during model selection.

**Figure 1:**
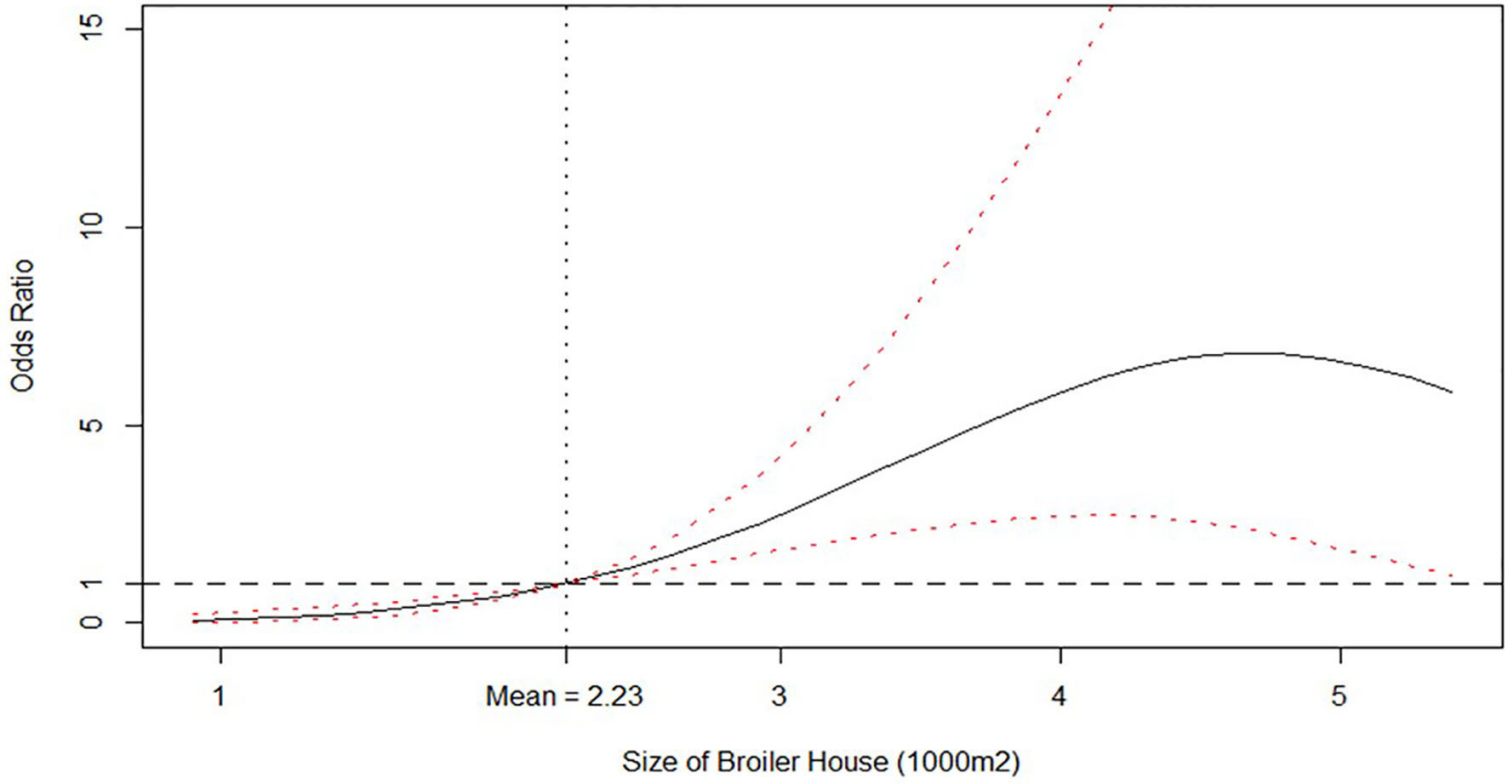
Odds ratio relationship between size of broiler house (1,000 m^2^) and Probability of isolating *Salmonella* spp. from litter. Odds ratio is relative to the mean value, which is shown by the vertical dashed line. Solid line is the posterior median odds ratio and red dashed lines are 95% credible intervals. Horizontal dashed line shows odds ratio = 1 for reference.

After excluding insignificant covariates shown in Table 3 and re-estimating the model from equation (1), the DIC from Table 4 indicates that the new specification is indeed preferred over the latter (530.01 for model from Table 4 vs. 535.97 for model from Table 3). Table 4 reports that a quadratic effect between the size of broiler house and the number of litter reutilizations were identified but with opposite responses: size of broiler house was found to increase the odds of isolating *Salmonella* spp. in litter, peaking for broiler houses between 4,000 and 5,000 m^2^ and decreasing thereof. Notice that the average size is 2,230 m^2^. This effect is better observed from Figure 1, where the posterior distribution of the calculated Odds Ratio (O.R.) of size of broiler house, with respect to the mean value, is graphed.

**Table 4:** Bayesian hierarchical logit posterior medians and credible intervals including only significant covariates and specific random effects.

**Variable**	**Parameter**	**Median**	**2.5%**	**97.5%**	**Geweke^**a**^**
Intercept	β_0_	−2.427	−4.285	−0.685	0.9
House size	β_1_	2.921	1.385	4.541	−0.9
House size^2^	β_2_	−0.310	−0.543	−0.077	0.9
Litter_use	β_3_	−0.227	−0.458	−0.017	0.2
Litter_use^2^	β_4_	0.018	0.002	0.037	−0.1
Total housing size	β_5_	−0.281	−0.419	−0.159	0.6
Type2	β_6_	−1.154	−2.193	−0.200	0.6
Type3	β_7_	−1.921	−3.275	−0.697	0.8
Rearing cycle1	δ_1_	−0.518	−0.884	−0.124	−0.6
Rearing cycle2	δ_2_	0.189	−0.038	0.481	−0.3
Rearing cycle3	δ_3_	0.312	0.038	0.617	0.7
Spatial var1	$\tau_{1}^{2}$	0.005	0.001	0.028	0.5
Spatial var2	$\tau_{2}^{2}$	0.005	0.001	0.037	−0.3
Spatial var3	$\tau_{3}^{2}$	0.005	0.001	0.033	−1.1
Time var	$\tau_{T}^{2}$	0.113	0.010	0.807	0.0
Spatial autocorrelation	ρ_*S*_	0.224	0.011	0.691	0.7
Time autocorrelation	ρ_*T*_	0.380	0.021	0.896	0.3
	DIC^b^ = 530.01				

*Dependent variable is the isolation of Salmonella spp. in litter (n = 417)*.

a *Geweke diagnostic: values lower than |1.96| suggest good mixing of the chains*.

b *Deviance information criterion*.

**Figure 2:**
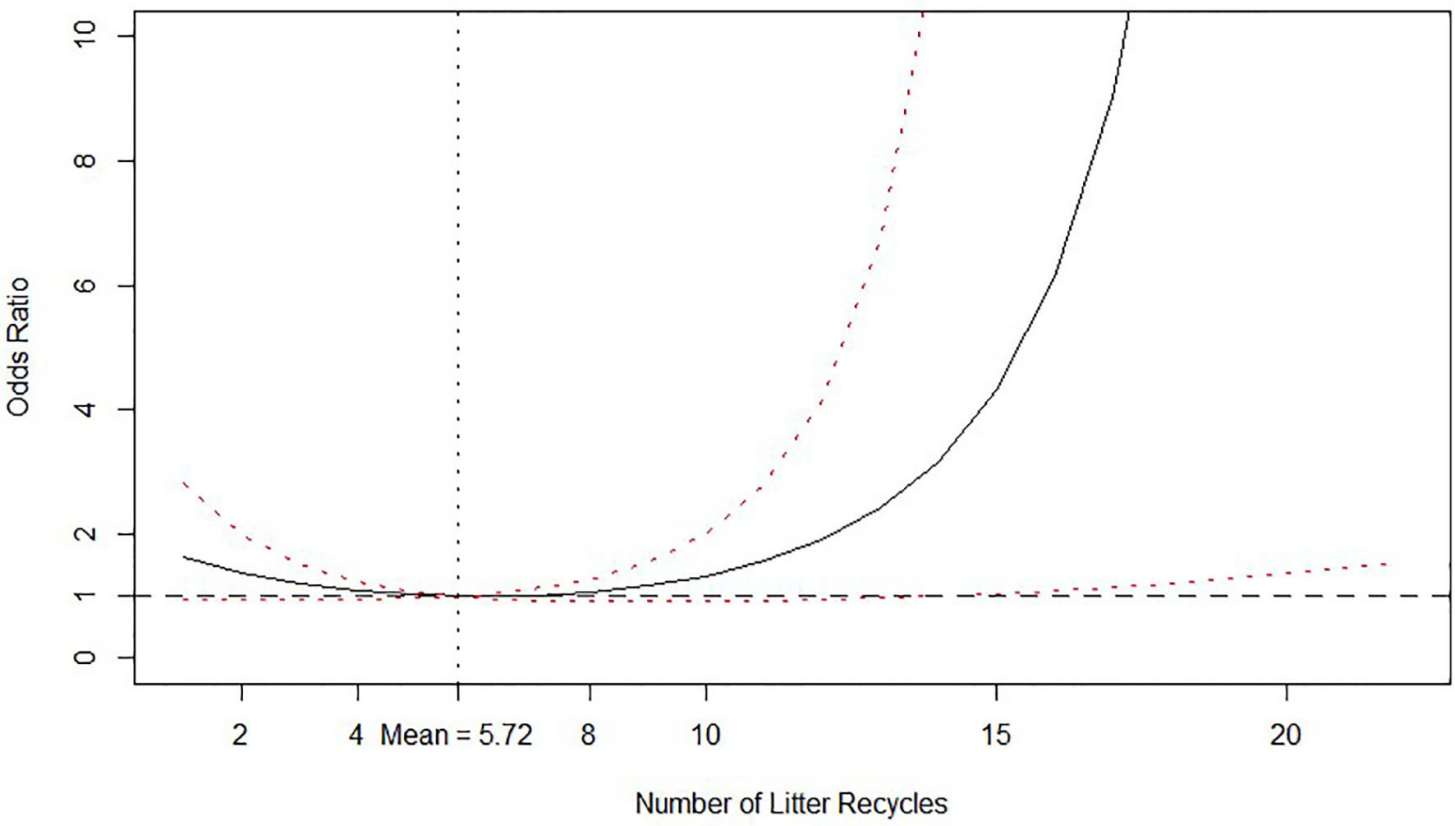
Odds ratio relationship between number of litter recycles and probability of isolating *Salmonella* spp. from litter. Odds ratio is relative to the mean value, which is shown by the vertical dashed line. Solid line is the posterior median odds ratio and red dashed lines are 95% credible intervals. Horizontal dashed line shows odds ratio = 1 for reference.

Number of litter recycles decreased the odds of isolating *Salmonella* spp. in litter up to five to six recycles and increased thereof, while the average number of recycles is 5.72 times. The posterior distribution with credible intervals of the calculated O.R. of number of litter recycles is graphed in Figure 2 for better reference.

**Figure 3:**
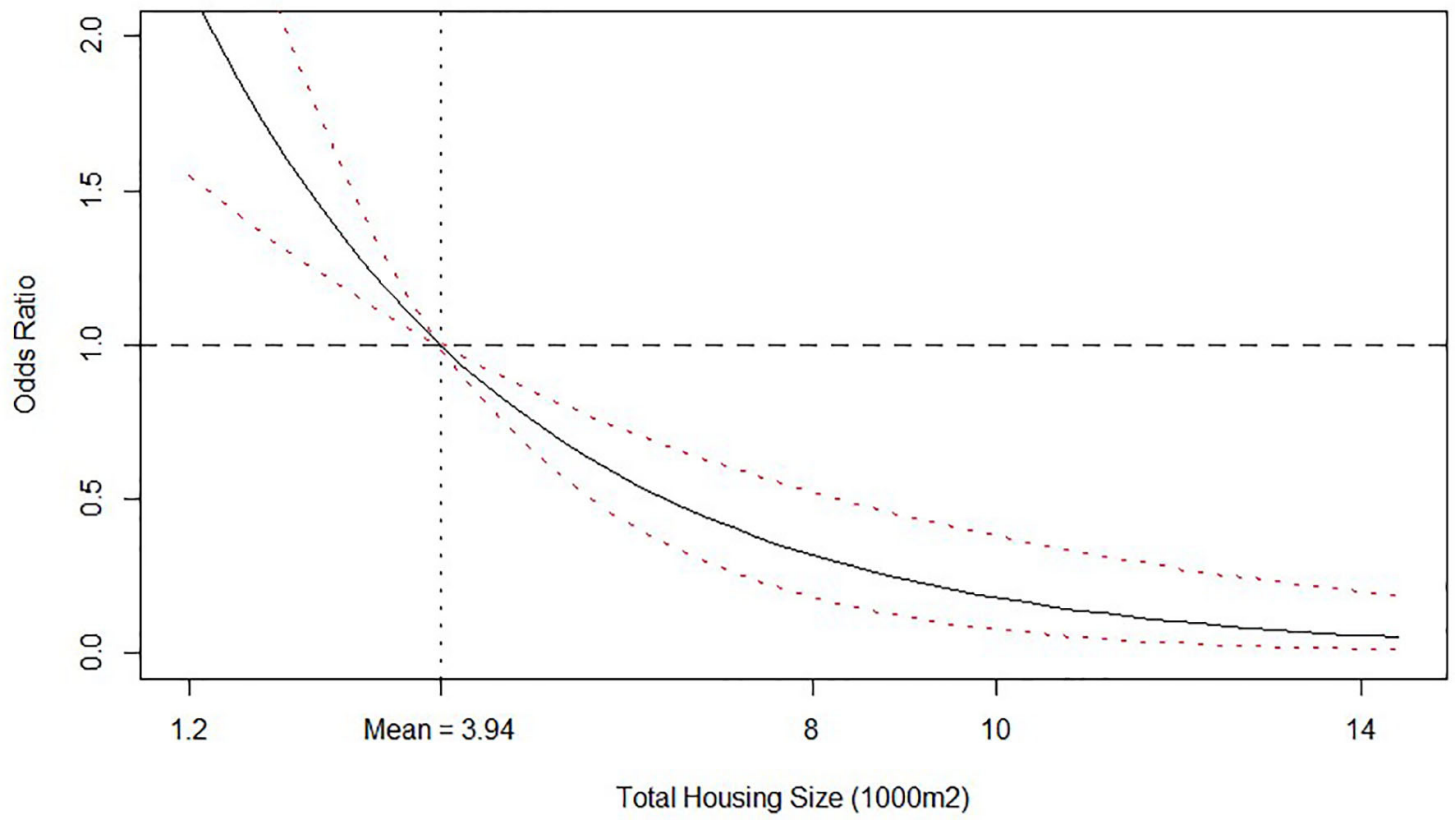
Odds ratio relationship between Total housing size (1,000 m^2^) and probability of isolating *Salmonella* spp. from litter. Odds ratio is relative to the mean value, which is shown by the vertical dashed line. Solid line is the posterior median odds ratio and red dashed lines are 95% credible intervals. Horizontal dashed line shows odds ratio = 1 for reference.

Total housing size had a linear negative effect on the log of odds of isolating *Salmonella* spp. in litter, as viewed by the negative value of the posterior median for this variable (Table 4). To better understand this effect, we calculate the posterior distribution and plot median values and credible intervals of the O.R. of the total housing size value with respect to the mean value (3,940 m^2^) and depict the response in Figure 3. It is clear that farms with bigger housing capacity, not necessarily bigger houses, are less likely to be tested positive for *Salmonella* spp. in litter than farms with smaller capacity. One possible explanation for this effect may be that farms with more housing area tend to be more specialized, leading to better management practices during and between the rearing period. Although technical support is provided by the integrator, every farmer is responsible for carrying out husbandry and disinfection procedures under regular supervision of a qualified technician, which can ultimately lead to differences not only on the odds of isolating *Salmonella* spp. but also on performance parameters^7^.

**Figure 4:**
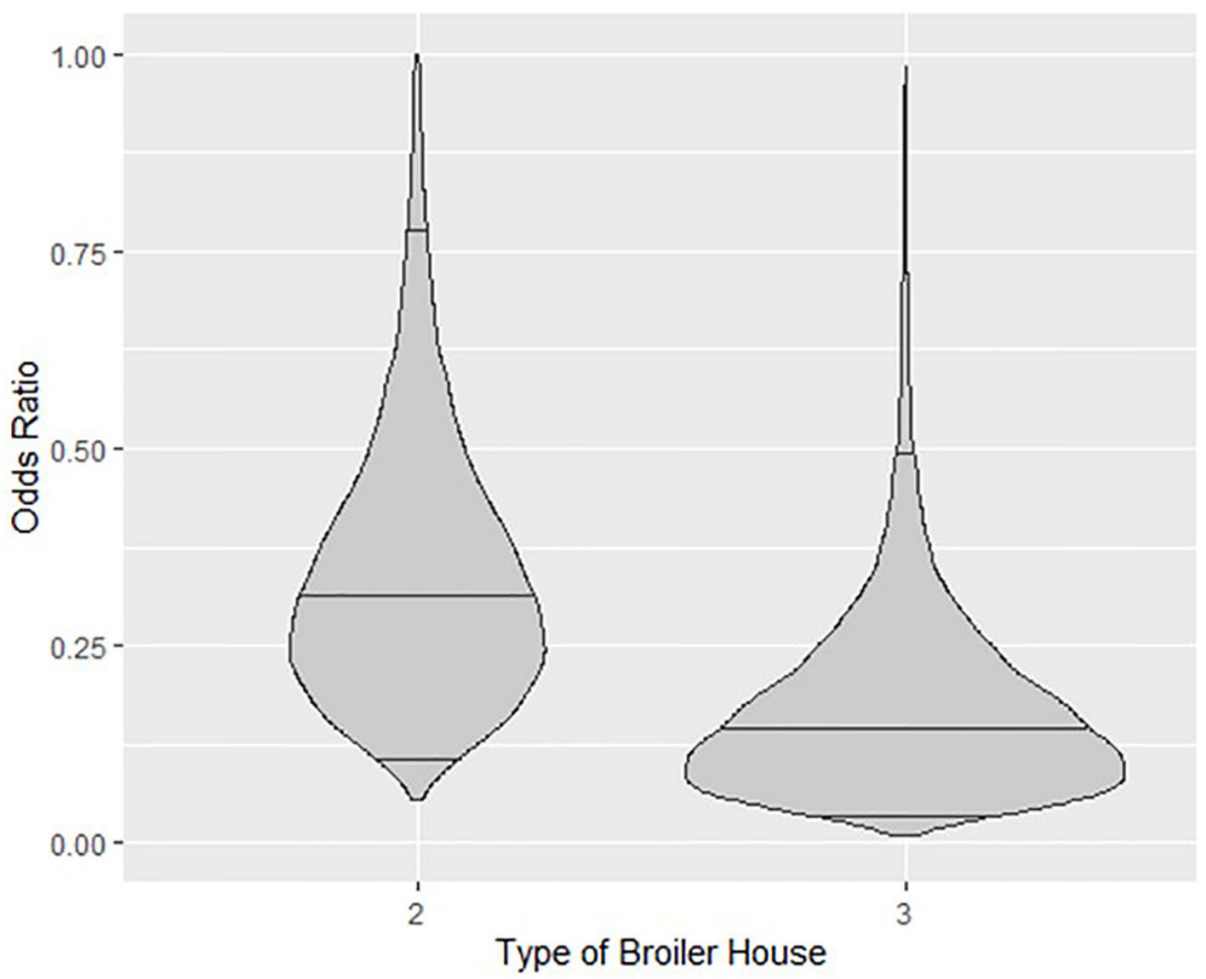
Violin plots showing the posterior density of the estimated Odds Ratio relationship between types of broiler house 2 and 3 with respect to type1 and probability of isolating *Salmonella* spp. from litter. Horizontal lines inside plots represent posterior medians and 95% credible intervals.

Categorical variables for broiler house type reduced the probability of detection of *Salmonella* spp. Odds ratio (O.R.) calculated for a type 2 broiler house reveals that the odds of isolating *Salmonella* spp. from litter of this type of building is 68% lower (O.R.≅0.32) than from a type 1 building, with credible intervals ranging from 18% (O.R.≅0.82) to 89% (O.R.≅0.11). Similarly, the odds of isolating *Salmonella* spp. from type 3 buildings is 85% lower (O.R.≅0.15), with credible intervals ranging from 50% (O.R.≅0.5) to 96% (O.R.≅0.04). These relationships are graphed in Figure 4, where the posterior distributions of the calculated O.R. with respect to type 1 houses, with median values and 95% credible intervals are shown in violin plots.

Regarding type of broiler house, in this study, conventional broiler houses (with lateral curtains used to control temperature and air flow) were classified into two categories: type 1 and type 2. The main difference attributed between both relates to the age of the building, that for type 1 houses was >5 years, while for type 2 houses was lower than 5 years. Type 3 houses, however, are broiler houses without curtains, but with evaporative cooling systems, which means that there is no direct contact with outdoor environment and the entrance of wild birds or rodents is markedly reduced.

**Figure 5:**
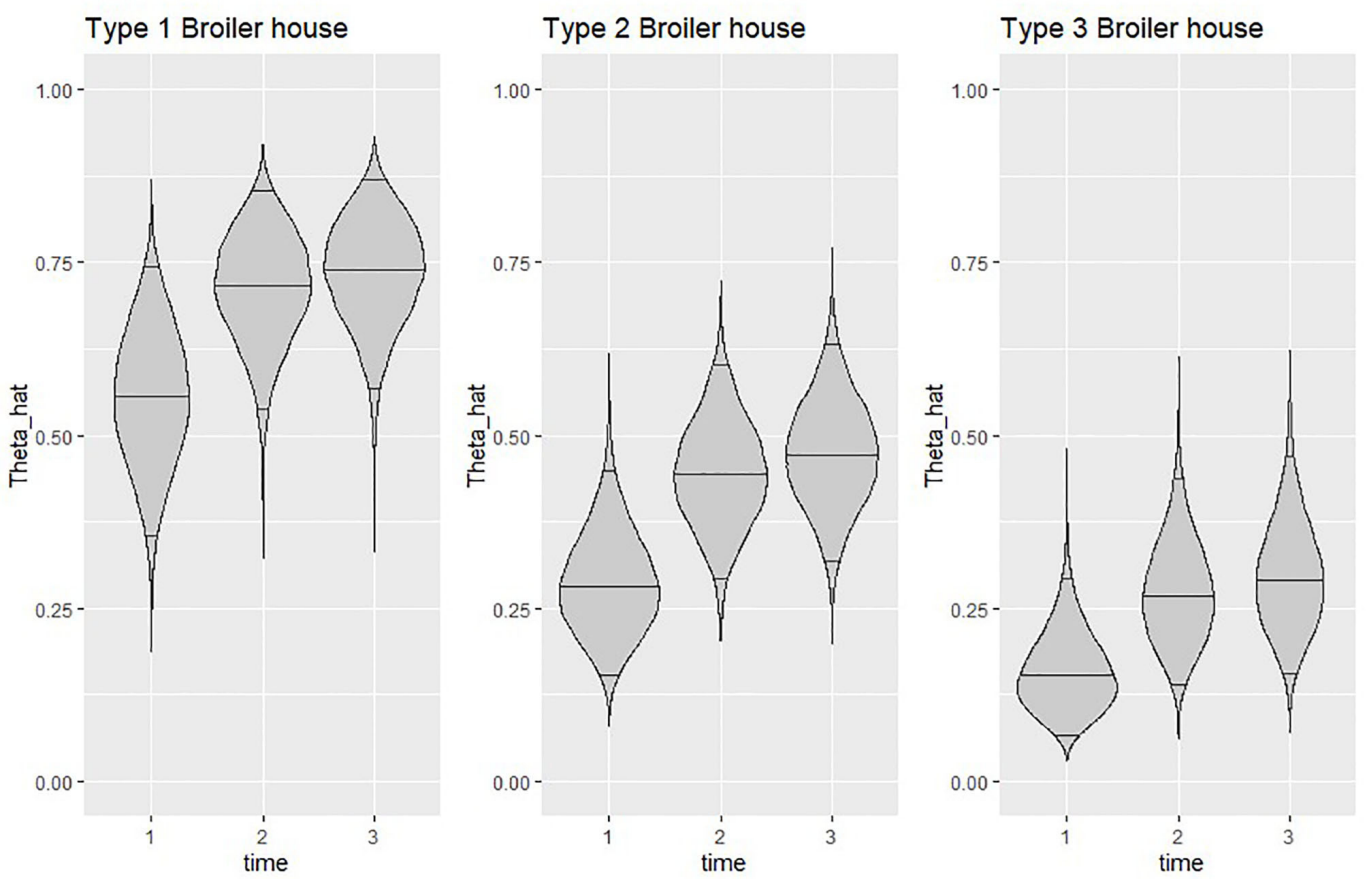
Violin plots showing the posterior density of the average estimated probability of isolating *Salmonella* spp. at the end of each time point for all types of broiler houses. Horizontal lines inside plots represent posterior medians and 95% credible intervals. $\text{Theta}\text{\_}\text{hat}=\hat{\theta_{\text{t}}}=\frac{\text{e}x\text{p}\left({\bar{\text{X}}}_{\text{t}}^{\text{T}}\beta +\delta_{\text{t}}\right)}{1+\text{e}x\text{p}\left({\bar{\text{X}}}_{\text{t}}^{\text{T}}\beta +\delta_{\text{t}}\right)},for\;{\bar{\text{X}}}_{\text{t}}=\frac{\overset{\text{K}}{\underset{\text{k}=1}{\sum }}{\text{X}}_{\text{k}\text{t}}}{\text{K}}$.

The estimated time specific effects (δ_*t*_) revealed a positive trend on the probability of isolating *Salmonella* spp. in litter, as observed on the graphical representation from Figure 5 of the estimated probability of isolating *Salmonella* spp. in litter for each type of broiler house across the evaluation period.

Figure 5 clearly shows a similar increase in estimated probability of isolating *Salmonella* spp. from litter of all types of broiler houses, but also highlights the difference in probability between houses, which seems to remain similar throughout the study. When looking at posterior median, type 3 broiler houses calculated probabilities were 60–70% lower than type 1, while calculated probabilities for type 2 houses were 36–50% lower than type 1.

Posterior median of correlation coefficients and variances (Table 4) show evidence of positive spatial autocorrelation (ρ_*S*_ = 0.2247) and time autocorrelation (ρ_*T*_ = 0.3808). Estimates for spatial variation for every time period ($\tau_{t}^{2}$) were very similar, suggesting no significant differences on variance of the probability of detection of *Salmonella* spp. across space.

For comparison purposes, we show the covariate estimates without accounting for temporal or spatial autocorrelation in Table 5. Although the comparison of Bayesian estimates with estimates obtained using the frequentist approach are not appropriate, we observe that estimated coefficients were overall smaller than the obtained posterior medians when assuming residuals are i.i.d, and the coefficients for litter recycles were only marginally significant. This will carry much uncertainty on the determination of relevant risk factors and especially for the case of litter recycles, will lead to unreliable standard errors and consequent estimation of confidence intervals for O.R.

**Table 5:** Logit estimates with covariates and interaction terms without accounting for spatial and temporal effects.

**Covariate**	**Estimate^**a**^**	**Std. error**	***p*-value**
Intercept	−2.263**	0.912	0.013
House size	2.723***	0.779	0.001
House size^2^	−0.290**	0.115	0.012
Total housing size	−0.263***	0.063	<0.001
Type2	−1.055**	0.479	0.027
Type3	−1.785***	0.625	0.004
Litter_use	−0.186*	0.109	0.086
Litter_use^2^	0.014*	0.008	0.097

*Dependent variable is the isolation of Salmonella spp. in litter (n = 417)*.

a *Maximum likelihood estimation obtained under the generalized linear model framework with logit link function*.

*** *Denote significance at the 1% level*.

** *Denote significance at the 5% level*.

* *Denote significance at the 10% level*.

Regarding cost calculations for the economic analysis, Table 6 reports the calculated posterior medians and 95% credible intervals for the probabilities of isolating *Salmonella* spp. from litter according to the number of litter recycles. The calculated probabilities in Table 6 are consistent with the O.R. relationship depicted in Figure 2. It is clear from Table 6 and Figure 2 that setting the number of recycles in six provides the lowest probability of isolating *Salmonella spp*.

**Table 6:** Posterior medians and credible intervals for the calculated probabilities of isolating *Salmonella* spp. from litter according to the number of litter recycles (*n* = 15,000 samples).

**Number of recycles**	**Median**	**2.5%**	**97.5%**
1	0.448	0.396	0.496
2	0.406	0.316	0.494
3	0.374	0.260	0.495
4	0.351	0.222	0.497
5	0.337	0.199	0.497
6	0.333	0.188	0.501
7	0.336	0.187	0.509
8	0.347	0.196	0.519
9	0.367	0.213	0.555
10	0.397	0.239	0.585
11	0.437	0.270	0.626
12	0.488	0.303	0.682

Table 7 reports the cost calculations related to litter recycles for each type of broiler house evaluated in this study, and reported in Miele et al. (37). Costs and returns are expressed as a proportion of total working cost per broiler house. Expected loss from positive flocks shows how much discount would be applied to those producers who decided to recycle litter beyond six periods.

**Table 7:** Calculated costs of litter replacement per flock, expected returns, gains, losses, and net return for each type of broiler house according to the number of litter recycles.

**Type of broiler house**	**Cost/flock of litter replacement^**a**^ %**	**Number of recycles**	**Expected return^**b**^ %**	**Expected loss from positive flocks^**c**^ %**	**Expected net return^**d**^ [min %, median %]**
Type 1	12.32	6 (baseline)	14.25	–	[14.25, 14.25]
	11.91	7	14.65	1.97	[10.56, 12.68]
	10.59	8	15.97	2.22	[11.37, 13.75]
	9.57	9	17.00	2.50	[11.97, 14.50]
	8.74	10	17.82	2.83	[12.42, 14.99]
	8.07	11	18.49	3.23	[12.68, 15.26]
	7.51	12	19.05	3.72	[12.74, 15.33]
Type 2	11.73	6 (baseline)	15.18	–	[15.18, 15.18]
	11.34	7	15.57	2.09	[11.28, 13.48]
	10.08	8	16.83	2.34	[11.98, 14.49]
	9.10	9	17.81	2.61	[12.54, 15.19]
	8.32	10	18.59	2.95	[12.95, 15.64]
	7.68	11	19.23	3.36	[13.19, 15.87]
	7.15	12	19.76	3.86	[13.22, 15.90]
Type 3	14.61	6 (baseline)	15.36	–	[15.36, 15.36]
	13.10	7	16.86	2.27	[12.15, 14.59]
	11.54	8	18.43	2.56	[13.12, 15.87]
	10.32	9	19.65	2.88	[13.84, 16.76]
	9.35	10	20.62	3.27	[14.37, 17.35]
	8.55	11	21.42	3.74	[14.69, 17.67]
	7.89	12	22.08	4.31	[14.77, 17.77]

a *Cost estimated as a percentage of total working cost for each type of broiler house*.

b *Expected return calculated considering total capital cost and a 6% annual rate, and expressed as a percentage of total working cost according to the type of broiler house*.

c *Expected loss from positive flocks calculated by the product of the posterior median of the probability of isolating Salmonella (percentage of positive flocks) and the 40% revenue discount for positive flocks*.

d *Posterior distribution of net returns obtained by subtracting the expected return and the expected Loss related to litter recycles. Minimum and Median values are displayed*.

For our example, we see that by recycling litter for an additional six cycles, the producer will be able to dilute the cost with litter replacement into 12 cycles (including first six cycles to which no penalty was applied) Such distribution of costs is clear when we observe cost/flock of litter replacement, which decreases for all types of houses, but is numerically greater for type 3 houses (column 2 from Table 7). The different cost structures for each broiler house indicates that there might be different incentives to recycle litter. Table 8 shows that for broiler houses of type 1 and 3, using a discount rate of 1% per cycle, and considering the baseline scenario as $100 expected payment per flock for each type of broiler houses, producers will choose to extend recycle until 12 rearing cycles, as the calculated NPV, will be >12 equal payments. This decision will maximize expected NPV of producers but will also lead to a significant increase on the probability of detecting *Salmonella* spp. from litter.

**Table 8:** Net present value calculated for expected returns obtained for each type of broiler house.

**Type of broiler house**	**Number of recycles**	**Expected net Return^**a**^ %**	**Expected net return^**b**^ ($)**	**NPV (baseline)^**c**^**	**NPV (baseline + recycles)^**d**^**
Type 1	6 (baseline)	14.25	100	$1,125.51	$1,131.38
	7	12.68	88.98		
	8	13.75	96.49		
	9	14.50	101.75		
	10	14.99	105.19		
	11	15.26	107.08		
	12	15.33	107.58		
Type 2	6 (baseline)	15.18	100	$1,125.51	$1,121.95
	7	13.48	88.80		
	8	14.49	95.45		
	9	15.19	100.06		
	10	15.64	103.03		
	11	15.87	104.54		
	12	15.90	104.74		
Type 3	6 (baseline)	15.36	100	$1,125.51	$1,171.36
	7	14.59	94.98		
	8	15.87	103.32		
	9	16.76	109.11		
	10	17.35	112.95		
	11	17.67	115.04		
	12	17.77	115.69		

a *Median value of expected net return described in Table 7*.

b *Expected return in monetary terms assuming baseline value as $100*.

c *Net present value calculated using a discount rate of 1% per period and 12 equal payments of $100*.

d *Net present value calculated using a discount rate of 1% per period, 6 equal payments of $100 and the expected monetary returns depicted in column 4 for each type of broiler house*.

## Discussion

Size of broiler house, also named house area in other studies (17, 22, 38), significantly influenced probability of detection of *Salmonella* spp. This covariate did not significantly affect the response in those studies, while it was linked to increase in O.R. in other studies in laying hens (8, 39). From a transmission perspective, it might be possible that bigger houses, housing a greater number of birds, would be more likely to, given a potential contamination, favor pathogen amplification. In the present study, although density could not be effectively recorded, the same average number of birds per square meter are housed for different types and sizes of broiler houses^8^ in the enterprise. Furthermore, interactions between type and size of broiler house did not reveal significant effects, reducing the likelihood of a potential confounding between these variables.

Observations of reduced risk of *Salmonella* spp. positive flocks in the current study related to type of broiler house could be due to more stable and isolated environments on broiler houses of types 2 and 3. Such isolation is expected to reduce contamination from external sources vectored by birds, rodents or dust, which are constantly pointed as risk factors for *Salmonella* spp. contamination (6, 10, 35). This fact, linked to a potential greater commitment of the integrated producer on a higher fixed investment may be an explanation for the observed effect and may also explain the difference on O.R. between old and new buildings (types 1 and 2, respectively). Old buildings and old equipment are harder to disinfect, as they become worn out and with fixtures, favoring the accumulation of dirt, litter, and feces. Such effects, linked to a higher need for maintenance (replacing curtains, nets, disabling, and cleaning equipment) could lead to both an increased persistence of contamination, as well as an increased susceptibility for contamination from external sources (17, 40, 41). A similar explanation applies for the effect of total housing size and was already discussed.

When interpreting the difference in probabilities of detecting *Salmonella* spp. from different types of broiler houses across time, we see that although an overall increase on probabilities was observed, types 2 and 3 houses were relatively less affected than type 1. This finding indicates that one possible measure taken by the integrator to significantly reduce *Salmonella* spp. prevalence and potential losses at the end of the production chain will be to incentivize contracted producers to invest in new broiler houses or eventually contract with producers who have types 2 and 3 broiler houses.

Regarding Litter reutilization, it may be classified as a factor affecting persistence of contamination, because every broiler house's litter is commonly treated inside it in the between-flock period and hardly is exchanged with other broiler houses, even when in the same farm. The practice of recycling litter is common in Brazil due to the limited supply of wood shavings (the most common bedding material) and due to the high economic cost of replacing litter at the end of each cycle.

Persistence of *Salmonella* spp. in litter is well studied (9, 42) and known to be affected by moisture levels, temperature and ammonia levels during fermenting or composting, so that in aged litter (more recycled) higher levels of these factors are required to properly eliminate the bacterium (43–45). This behavior is reflected in the O.R. obtained for litter recycle, where a reduction on O.R. was observed with a subsequent increase. The initial reduction may be due to interactions of *Salmonella* spp. with other microorganisms colonizing litter in the early reutilizations: as less stablished is the microbiome of the litter, the less effect of competitive exclusion is observed, accounting for a relative higher presence of *Salmonella* spp. on first recycles, summed to the impacts of fermentation. At a certain point, however, fermentation starts to lose efficiency and persistence of *Salmonella* spp. is encouraged, as demonstrated in Kim et al. (45).

### Implications of Research Findings

Using a spatio-temporal Bayesian hierarchical binomial logistic regression model, this study shows that the probability of detecting *Salmonella* spp. in litter of broiler houses in the grow-out period is significantly affected by size of broiler house, total housing area, type of broiler house, and number of litter recycles. To the author's best knowledge, it is the first study to evaluate risk factors related to *Salmonella* spp. isolation in broiler chicken litter in Brazil, and also to use data routinely and consistently collected by a broiler enterprise. Some authors assess the prevalence of specific Salmonella serotypes in the same region, but use pooled data (without accounting for spatial or time autocorrelation) from different enterprises and report values between 5 and 11% (46–48). More recently, Voss-Rech et al. (42) evaluating nine broiler houses in the same region, showed that non-typhoidal Salmonella persisted in contaminated farms but did not link the results to risk factors.

In our study, covariates were selected according to the classification used by the enterprise, which may have aggregated various factors affecting *Salmonella* transmission or persistence into one variable. This classification, however, is made according to several requirements on standard biosecurity practices, and potential variations on these factors would be exceptions to the established requirement. Therefore, the covariates adopted in this study are effectively eligible to be changed by the enterprise, although some variables such as type and size of broiler houses may be more difficult to change than others like litter recycle.

Economic loss of having positively tested flocks should also be considered when setting strategies to reduce prevalence of the bacterium. If a flock tests positive for *Salmonella* spp. 2 weeks before slaughter, this flock will be processed differently at the slaughterhouse to reduce the risk of carcass contamination (25) and cannot be used to manufacture of products with more added value (processed products) but mostly directed to fresh or frozen products. This leads to revenue loss for the integrator as well as an increase in costs because *Salmonella* spp. positive flocks sometimes are held at the farm to be slaughtered at the end of the day to minimize risk of infecting negative flocks. Depending on the prevalence recorded for the integrator, flocks can be held even until one determined day (e.g., end of a given week), leading to significant increases in feed costs. Following the directives of the ministry of agriculture for the surveillance and control of *Salmonella*, based on World Organization for Animal Health [OIE] (49), every flock must be surveilled at least once before slaughter, and this information is further used for risk assessment by the veterinary authority. Therefore, data on *Salmonella* spp. occurrence is available for the integrator but may not be always stored in a way that allows effective data analysis, or is misused in terms of risk assessment.

With a proper system of data collection and an accurate model, it is possible to estimate the potential losses arising from *Salmonella* spp. contamination, unrelated to foodborne diseases. The detection on the pre-slaughter period and adoption of control measures markedly decreases this risk (25), but the costs of implementing different processing strategies, slaughter segregation, and restricted access to different markets have not been defined. To the author's best knowledge, there is no such a description in literature.

Using available cost information and estimated probabilities of detecting *Salmonella* spp. from litter related to litter recycles, we estimated how costs on replacing litter and its impact on producers profitability would incentivize producers working with type1 and type 3 broiler houses to use litter beyond the recommended number of recycles, which would lead to an increase in the odds of positive flocks. We also reported in Table 7 the minimum expected net return per flock, calculated using the upper limit of probability of detecting *Salmonella* spp. related to litter use to allow for comparisons on the process of decision making under extreme risk aversion from producers (although we didn't formally account for risk in our analysis).

From a min-max selection criteria, considering that producers will choose to have the best possible performance in the worst case (50), the expected net return for all subsequent cycles after cycle number six is lower than the baseline scenario. This would discourage risk-averse producers to recycle litter with the objective of maximizing expected returns.

However, as this kind of extreme risk aversion does not always hold (51), it is possible that some producers would chose to maximize NPV, using the posterior median of expected returns as an estimate of payments, and ignore the issue of return variation. NPV analysis discounts a future stream of returns to compare decisions that may remain unchanged by decision makers for multiple rearing cycles. Under this decision rule, the problem faced by the producer is to choose between 12 consecutive payments equal to the baseline scenario, or 6 baseline consecutive payments followed by six variable payments according to each expected return.

This simple example highlights the importance of defining the risk factors related to *Salmonella* spp. occurrence and its respective cost share to allow effective control strategies and explains why producers may choose strategies that would lead to greater risk of *Salmonella* spp. occurrence. It is interesting to note that type 1 broiler houses were characterized in this study as riskier than types 2 and 3 with respect to *Salmonella* spp. isolation, and the economic incentive to recycle more litter may bring even more risk to the enterprise. Similarly, while type 3 broiler houses owners are incentivized to recycle more litter, this type of broiler house had the lowest probability of being contaminated with the bacterium.

The presented result is highly dependent on how the decision maker parametrizes the problem, in terms of assumption of decision rules (risk aversion), cost determination, cost share, and incentives. If the penalty applied for positive flocks is greater, the result will be different (in favor of adopting the proposed replacement scheme), as well as if instead of a penalty, a premium is paid for negative flocks, especially on the first six cycles period (aiming to reduce contamination or persistence of the bacterium), the risk for litter recycle on *Salmonella* spp. isolation may be reduced. These types of incentives clearly relate to an attempt to solve the principal-agent problem that is frequently described in agricultural cooperatives (52), as the producer and the enterprise manager may not share the same objective, which in this case is minimize *Salmonella* spp. occurrence. In this regard, there will be a conflict of interest between the enterprise (the principal) and the producer (the agent), in which the first maximizes profit by having the lowest possible rate of *Salmonella* spp. positive flocks, while the second has an incentive to maximize profit by reducing variable production costs, which may increase the rate of positive flocks. This may imply on a different optimal solution for the principal and the agent, creating the principal-agent problem. Although we didn't formally analyze the problem, specifying a function to be optimized by the principal, our study suggests that it is essential for the enterprise to establish clear contract terms to avoid asymmetric information and incentivize producers to adopt measures that will lead to a reduction on *Salmonella* spp. occurrence.

Our study is also important to shed light on the benefits for the enterprise of using official data linked to a systematic classification of broiler farms to identify risk factors related to occurrence of *Salmonella* spp., the importance of accurate cost determination and the use of incentives to induce producers adopting procedures related to the elimination of the bacterium. The advantages for the enterprise include understanding the probable causes of outbreaks and, given a more detailed follow up, the costs and benefits involved in prevention and control of the infection and the adoption of optimal control strategies.

## Conclusions

This longitudinal study is the first Brazilian study using official data recorded from a broiler enterprise to establish risk factors related to farm characteristics and management strategies affecting the probability of isolating *Salmonella* spp. at the end of the grow-out period. We show evidence of spatial and time autocorrelation, which were accounted for by means of a Bayesian hierarchical model. Factors potentially related to the horizontal transmission of Salmonella, like type of broiler house, size of broiler house and total housing size significantly affected the probability of isolating the bacterium in litter. The number of litter recycles, likely related to the persistence of infection within broiler houses, also affected such probability.

We show how the risk for *Salmonella* spp. isolation increases as each of the risk factors change and we give an example where the producers will chose litter recycles strategies that will lead to increased probability of *Salmonella* spp. occurrence and discuss the role of establishing economic incentives to avoid the principal-agent problem and reduce the risk for positive flocks. Although the modeled scenarios may vary according to the cost and incentives adopted, it potentially shows an example of principal–agent problem and how it may impact *Salmonella* spp. persistence in the enterprise.

Future studies including more cycles and different covariates may clarify the dynamics of bacterial spread and allow for the establishment of optimal control strategies. Relationship of *Salmonella* spp. presence and production performance may also help clarify the effect of house size and farm capacity while allowing for a more accurate calculation of costs and returns for each evaluated farm.

Our study sheds light on the importance to use official data and systematic classification of farms and broiler houses to define risks for the isolation of *Salmonella* spp. using a reliable model specification. Extending data collection and using it to parameterize a diffusion model is a promising alternative for the enterprise to establish optimal control measures.

## Data Availability Statement

The datasets presented in this article are not readily available because the data sets generated for this study will not be made publicly available. Identification of farms and spatial locations are sensitive information from the enterprise and cannot be disclosed. Data on bacterial isolation may be disclosed upon request. Requests to access the datasets should be directed to pedrocm@okstate.edu.

## Ethics Statement

Data on bacterial isolation from farms was provided by the enterprise and was collected as routine for official notification. Therefore, all data reported results from commercial broiler production, which is regulated by the Brazilian Ministry of Agriculture. Approval of an ethic committee is not required for the study type.

## Author Contributions

PM and CC contributed conception and design of the study. PM organized the database and performed the statistical analysis. PM wrote the first draft of the manuscript. All authors contributed to manuscript revision, read, and approved the submitted version.

## Conflict of Interest

The authors declare that the research was conducted in the absence of any commercial or financial relationships that could be construed as a potential conflict of interest.

## Footnotes

^1^The cutoff value of 20 km will measure how many broiler houses are located within up to 20 km of other broiler houses. On average, the distance between broiler houses was 11.45 km.

^2^Total working costs are defined by the author as the sum of labor, litter, wood, and electricity, maintenance, insurance, propane, paper for housing chicks, quick lime, extras (including other utilities), depreciation, and environmental costs (licenses).

^3^This cost includes previously reported costs plus investment in buildings and equipment.

^4^The enterprise does not have any policy to implement discounts for positive flocks or underperformance. 40% discount was arbitrarily selected to imply a severe penalty.

^5^DIC for model including interactions was 549.66.

^6^The quadratic functional form was also tested for number of broiler houses and total housing size but was not preferred over the linear functional form. DIC for model presented in Table 3, including quadratic terms for all numeric variables was 536.46. For brevity, estimates are not shown in Table 3.

^7^Data related to performance parameters, like feed efficiency, daily gain, and mortality could help clarifying the reason why farms with larger housing area, but not necessarily with more houses, were less likely to have the bacterium isolated from litter. However, due to confidentiality issues, this data could not be provided.

^8^Normally, type 3 broiler houses, which have a controlled environment and more stable temperature and moisture conditions tend to accommodate higher densities, but this information was not fully disclosed for this study.
